# Longer apneas and hypopneas are associated with greater ultra-short-term HRV in obstructive sleep apnea

**DOI:** 10.1038/s41598-020-77780-x

**Published:** 2020-12-09

**Authors:** Salla Hietakoste, Henri Korkalainen, Samu Kainulainen, Saara Sillanmäki, Sami Nikkonen, Sami Myllymaa, Brett Duce, Juha Töyräs, Timo Leppänen

**Affiliations:** 1 Department of Applied Physics, University of Eastern Finland, Kuopio, Finland; 2 Diagnostic Imaging Center, Kuopio University Hospital, Kuopio, Finland; 3 Sleep Disorders Centre, Department of Respiratory and Sleep Medicine, Princess Alexandra Hospital, Brisbane, Australia; 4 Institute for Health and Biomedical Innovation, Queensland University of Technology, Brisbane, Australia; 5 School of Information Technology and Electrical Engineering, The University of Queensland, Brisbane, Australia

**Keywords:** Respiratory tract diseases, Sleep disorders

## Abstract

Low long-term heart rate variability (HRV), often observed in obstructive sleep apnea (OSA) patients, is a known risk factor for cardiovascular diseases. However, it is unclear how the type or duration of individual respiratory events modulate ultra-short-term HRV and beat-to-beat intervals (RR intervals). We aimed to examine the sex-specific changes in RR interval and ultra-short-term HRV during and after apneas and hypopneas of various durations. Electrocardiography signals, recorded as a part of clinical polysomnography, of 758 patients (396 men) with suspected OSA were analysed retrospectively. Average RR intervals and time-domain HRV parameters were determined during the respiratory event and the 15-s period immediately after the event. Parameters were analysed in three pooled sex-specific subgroups based on the respiratory event duration (10–20 s, 20–30 s, and > 30 s) separately for apneas and hypopneas. We observed that RR intervals shortened after the respiratory events and the magnitude of these changes increased in both sexes as the respiratory event duration increased. Furthermore, ultra-short-term HRV generally increased as the respiratory event duration increased. Apneas caused higher ultra-short-term HRV and a stronger decrease in RR interval compared to hypopneas. In conclusion, the respiratory event type and duration modulate ultra-short-term HRV and RR intervals. Considering HRV and the respiratory event characteristics in the diagnosis of OSA could be useful when assessing the cardiac consequences of OSA in a more detailed manner.

## Introduction

Obstructive sleep apnea (OSA) affects approximately 1 billion people worldwide^1^, thus being one of the most prevalent sleeping disorders. The severity of OSA is assessed predominantly by the apnea-hypopnea index (AHI) determined from polysomnography (PSG)^2^. While the electrocardiography (ECG) is always recorded during a PSG, and it is often measured with a Home Sleep Apnea Test, neither ECG nor heart rate variability (HRV) parameters are systematically utilized in current OSA diagnostics. Short-term (~ 5 min) and long-term (~ 24 h) ECG measurements are considered the most suitable options when clinically assessing HRV^3,4^. However, the suitability of ultra-short-term (< 5 min) HRV analysis has also been noted^3,5^.

HRV, the beat-to-beat oscillations of the RR interval, is generated by the autonomic nervous system (ANS) and neuro-cardiac interactions^3^. The sympathetic and the parasympathetic nervous systems (SNS and PNS) work together to maintain the homeostasis of the body: normally, the SNS reacts rapidly to stress whereas the PNS is dominant in rest^6,7^. Higher SNS activity is associated with low long-term HRV and shorter RR intervals whereas higher PNS activity associates with high long-term HRV and longer RR intervals^7^. The proportion of SNS and PNS activities vary naturally during sleep depending on the sleep depth causing changes in heart rate^8^.

In OSA, abnormal respiratory events cause intermittent hypoxemia, hypercapnia, recurrent arousals, and intra-thoracic pressure swings leading to elevated SNS activity and decreased long-term HRV^9–11^. Increased SNS activity can further cause increased systemic inflammation, oxidative stress, enhanced prothrombotic state, vascular dysfunction, and exacerbate coronary artery disease^11^. In the long term, these changes can lead to electrical and structural cardiac remodeling^12^ and thus, increase the risk of cardiovascular diseases, e.g. hypertension^13^, heart failure^14^, and cardiac arrhythmias^11^ in OSA patients.

Previous studies have shown that long-term HRV is reduced in OSA patients, even during daytime^15,16^. Daytime measurements have shown that RR intervals shorten along with an increase in OSA severity^17^. In their pioneer study (*n* = 8)^18^, Sola-Soler et al*.* observed that longer apneas cause a greater decrease in RR interval after the events. To examine the connection between HRV and the duration of the respiratory event, more comprehensive studies, which include a larger pool of patients, are necessary. Apneas cause deeper oxygen desaturations than hypopneas^19^, and more severe desaturations increase the risk of cardiovascular diseases^20,21^. However, the effect of the respiratory event type on HRV is not well known. Most studies investigating the connection between HRV and OSA have included considerably more or only men as subjects^16^. Therefore, considering the possible sex-related differences in OSA-related HRV is warranted. Although women have significantly lower long-term HRV, they have a lower risk of cardiovascular diseases and greater parasympathetic input to cardiac regulation than age-matched men^22^. Women also tend to have less severe OSA with shorter respiratory events^10^.

Although ultra-short-term HRV analyses have not yet been adopted as a regular tool in OSA diagnostics, they could provide valuable information about the immediate cardiovascular consequences of individual respiratory events. Ultra-short-term HRV analysis enables the assessment of the changes in RR intervals during and immediately after the respiratory events; this would be impossible with longer RR interval segments. Especially time-domain HRV parameters have shown promise in ultra-short-term HRV analysis, whereas frequency-domain HRV analyses usually require longer time scales^3,5^. Although ultra-short-term HRV has not been extensively studied in OSA patients, it could provide valuable insight into immediate cardiac consequences of individual respiratory events.

In this study, our main hypothesis was that more severe respiratory events cause higher ultra-short-term HRV and a greater decrease in post-event RR intervals. In this context, we consider apneas to be more severe events than hypopneas and longer events to be more severe than shorter ones. In addition, since men generally have more severe OSA, we hypothesize that the magnitude of these changes is greater in men. Therefore, we aimed to study whether the duration and the type of the respiratory event and the sex affect the ultra-short-term HRV parameters and the changes in RR interval during and after respiratory events.

## Results

### Population characteristics

The demographic data of the studied population is presented in Table 1. Men had significantly lower BMI but no statistically significant differences in comorbidities existed between the sexes. Men were diagnosed to have moderate or severe OSA more often and they also had more severe OSA as indicated by greater AHI, oxygen desaturation index, and respiratory event duration than women.

**Table 1:** Characteristics of the study population comprising suspected OSA patients. Values are presented as a median (interquartile range) for continuous variables and as a count (percentage) for discrete variables.

	All	Men	Women
**Number of patients**	758 (100%)	396 (52.2%)	362 (47.8%)
Non-OSA (AHI < 5)	133 (17.6%)	43 (10.9%)	90 (24.9%)*
Mild OSA (5 ≤ AHI < 15)	234 (30.9%)	99 (25.0%)	135 (37.3%)*
Moderate OSA (15 ≤ AHI < 30)	179 (23.6%)	103 (26.0%)	76 (21.0%)
Severe OSA (AHI ≥ 30)	212 (28.0%)	151 (38.1%)	61 (16.9%)*
AHI [events/h]	15.6 (6.8, 31.9)	22.5 (10.1, 42.0)	11.3 (5.0, 20.6)*
ODI_3%_ [desaturations/h]	15.0 (4.8, 37.4)	21.2 (7.1, 45.9)	9.9 (3.2, 27.0)*
Total sleep time [min]	314.3 (259.4, 362.5)	302.5 (254.8, 356.5)	323.3 (268.5, 367.5)
**Event duration [s]**			
All	22.8 (16.8, 31.5)	23.9 (17.6, 32.7)	21.2 (15.8, 29.7)*
Apneas	23.8 (17.5, 31.8)	24.8 (18.4, 32.5)	21.0 (15.8, 29.3)*
Hypopneas	22.6 (16.7, 31.5)	23.6 (17.4, 32.7)	21.2 (15.8, 29.7)*
**Number of events**			
All	38,247	22,893	15,354*
Apneas	7585	5489	2096*
10 to 20 s	2652 (35.0%)	1699 (31.0%)	953 (45.5%)*
20 to 30 s	2687 (35.4%)	2029 (37.0%)	658 (31.4%)*
Over 30 s	2246 (29.6%)	1761 (32.1%)	485 (23.1%)*
Hypopneas	30,662	17,404	13,258*
10 to 20 s	12,206 (39.8%)	6240 (35.9%)	5966 (45.0%)*
20 to 30 s	9876 (32.2%)	5816 (33.4%)	4060 (30.6%)*
Over 30 s	8580 (28.0%)	5348 (30.7%)	3232 (24.4%)*
Age [years]	54.2 (43.4, 64.5)	55.3 (43.4, 65.6)	52.9 (43.7, 63.5)
BMI [kg/m^2^]	34.3 (29.4, 40.4)	32.8 (28.2, 38.4)	36.2 (30.6, 42.7)*
**Comorbidities**			
Diabetes Mellitus, type 1	5 (0.7%)	3 (0.8%)	2 (0.6%)
Diabetes Mellitus, type 2	147 (19.4%)	76 (19.2%)	71 (19.6%)
Hypertension	312 (41.2%)	170 (42.9%)	142 (39.2%)
Smokers	131 (17.3%)	73 (18.4%)	58 (16.0%)

Information about comorbidities was obtained from patient records.

*OSA *obstructive sleep apnea, *BMI* body mass index, *AHI* apnea–hypopnea index, *ODI*_*3%*_ oxygen desaturation index based on AASM 2012 scoring criteria (desaturation ≥ 3%).

*Statistically significant difference (*p* < 0.001) between men and women. Mann–Whitney *U* test was used for continuous and χ^2^-test for discrete variables.

### HRV in men

The average within-event RR interval was mainly longer, and the post-event RR interval was shorter with longer apneas and hypopneas compared to shorter events (Tables 2 and 3). The within-event standard deviation of RR intervals (SD), root mean square of the successive differences (RMSSD), and proportion of successive RR intervals differing more than 50 ms (pRR50) increased with increasing apnea and hypopnea duration (Tables 2 and 3). The post-event SD, RMSSD, and pRR50 were higher in 20–30 s and > 30 s apneas and hypopneas compared to the 10–20 s apnea and hypopnea events (Tables 2 and 3), respectively. The relative difference between within- and post-event HRV parameter values was greater in shorter apneas (Table 2) and hypopneas (Table 3).

**Table 2:** Within- and post-event time-domain HRV parameter values in different apnea duration groups for men and women.

	Within-event			Post-event		
	Men	Women	*p*-value	Men	Women	*p*-value
**10–20 s**						
*n* (%)	1699 (31.0)	953 (45.5)	** < 0.001**	1699 (31.0)	953 (45.5)	** < 0.001**
Average RR Interval [ms]	950	931	0.508	883	894	**0.005**
SD [ms]	33	31	0.222	45	40	**0.008**
RMSSD [ms]	22	25	** < 0.001**	31	33	0.038
pRR50 [%]	0.0	5.9	0.019	10.5	11.8	0.041
**20–30 s**						
*n* (%)	2029 (37.0)	658 (31.4)	** < 0.001**	2029 (37.0)	658 (31.4)	** < 0.001**
Average RR Interval [ms]	947	1012*	** < 0.001**	872	919*	** < 0.001**
SD [ms]	44*	40*	0.035	54*	46*	0.021
RMSSD [ms]	28*	33*	**0.001**	34*	38*	0.016
pRR50 [%]	7.4*	11.1*	** < 0.001**	12.5*	15.4*	0.014
**Over 30 s**						
*n* (%)	1761 (32.1)	485 (23.1)	** < 0.001**	1761 (32.1)	485 (23.1)	** < 0.001**
Average RR Interval [ms]	954*	937^†^	0.636	842*^†^	854*^†^	**0.002**
SD [ms]	56*^†^	48*^†^	** < 0.001**	53*	46	**0.001**
RMSSD [ms]	33*^†^	35*	0.376	33*	31^†^	0.111
pRR50 [%]	9.1*^†^	11.4*	0.224	12.5*	11.1^†^	0.248

Medians of parameters were calculated from electrocardiogram recorded during apnea events (within-event) and within a 15-s segment following the apnea event (post-event). The *p*-values presented in the table denote the statistical significance of the differences between men and women; the *p*-values indicating statistically significant differences (*p* < 0.01) are bolded. Mann–Whitney *U* test was used for continuous and χ^2^-test for discrete variables.

*HRV* heart rate variability, *n* the number of apneas, *SD* standard deviation of RR intervals, *RMSSD* root mean square of successive differences, *pRR50* the number of adjacent RR intervals differing more than 50 ms divided by the total number of RR intervals during the apnea.

*Statistically significant difference (*p* < 0.01) compared to the 10–20 s apneas.

^†^Statistically significant difference (*p* < 0.01) compared to the 20–30 s apneas.

**Table 3:** Within- and post-event time-domain HRV parameter values in different hypopnea duration groups for men and women.

	Within-event			Post-event		
	Men	Women	*p*-value	Men	Women	*p*-value
**10–20 s**						
*n* (%)	6240 (35.9)	5966 (45.0)	** < 0.001**	6240 (35.9)	5966 (45.0)	** < 0.001**
Average RR Interval [ms]	911	867	** < 0.001**	863	834	** < 0.001**
SD [ms]	29	25	** < 0.001**	39	33	** < 0.001**
RMSSD [ms]	24	23	**0.001**	27	24	** < 0.001**
pRR50 [%]	0.0	0.0	** < 0.001**	6.7	5.0	** < 0.001**
**20–30 s**						
*n* (%)	5816 (33.4)	4060 (30.6)	** < 0.001**	5816 (33.4)	4060 (30.6)	** < 0.001**
Average RR Interval [ms]	955*	905*	** < 0.001**	896*	859*	** < 0.001**
SD [ms]	36*	31*	** < 0.001**	43*	37*	** < 0.001**
RMSSD [ms]	27*	26*	** < 0.001**	29*	26*	** < 0.001**
pRR50 [%]	6.1*	4.5*	** < 0.001**	7.7*	6.3*	** < 0.001**
**Over 30 s**						
*n* (%)	5348 (30.7)	3232 (24.4)	** < 0.001**	5348 (30.7)	3232 (24.4)	** < 0.001**
Average RR Interval [ms]	978*^†^	892*^†^	** < 0.001**	890*	835^†^	** < 0.001**
SD [ms]	43*^†^	34*^†^	** < 0.001**	45*^†^	37*	** < 0.001**
RMSSD [ms]	33*^†^	25*	** < 0.001**	29*	24^†^	** < 0.001**
pRR50 [%]	9.1*^†^	4.6*	** < 0.001**	7.7*	5.3^†^	** < 0.001**

Medians of parameters were calculated from electrocardiogram recorded during hypopnea events (within-event) and within a 15-s segment following the hypopnea event (post-event). The *p*-values presented in the table denote the statistical significance of the differences between men and women; *p*-values indicating statistically significant differences (*p* < 0.01) are bolded. The statistical significance of the difference in parameter values between men and women and between hypopnea duration groups were assessed by using the Mann–Whitney *U* test.

*HRV* heart rate variability, *n* the number of hypopneas, *SD* standard deviation of RR intervals, *RMSSD* root mean square of successive differences, *pRR50* the number of adjacent RR intervals differing more than 50 ms divided by the total number of RR intervals during the hypopnea.

*Statistically significant difference (*p* < 0.01) compared to the 10–20 s hypopneas.

^†^Statistically significant difference (*p* < 0.01) compared to the 20–30 s hypopneas.

**Figure 1:**
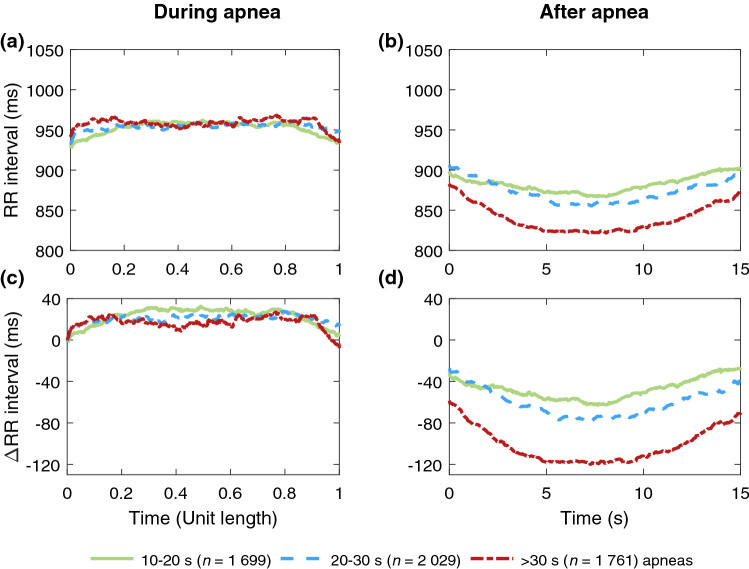
Median RR intervals of men (*n* = 321) (**a)** during apneas of different durations (*n* = 5 489) and (**b)** during a 15-s segment following apneas. The absolute change in RR intervals relative to the beginning of the event during and after apneas are presented in subfigures (**c)** and (**d)**, respectively. In subfigures (**a)** and (**c)** the duration of every apnea event is normalized with its duration.

**Figure 2:**
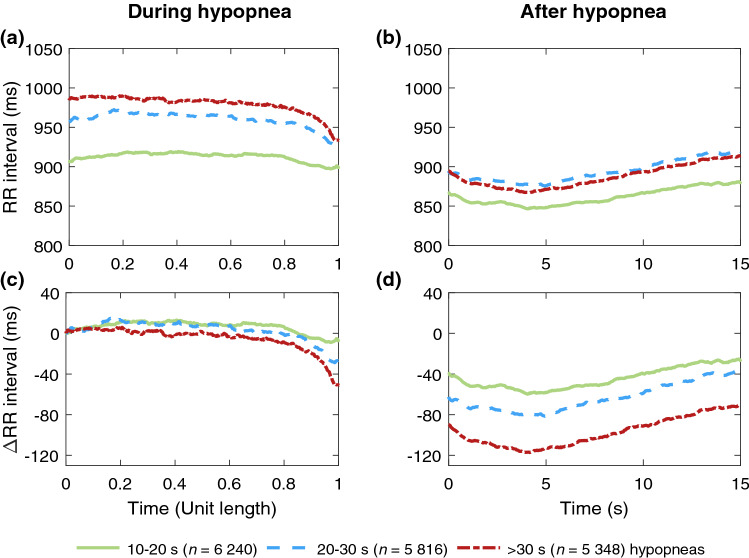
Median RR intervals of men (*n* = 395) (**a)** during hypopneas of different durations (*n* = 17 404) and (**b)** during a 15-s segment following hypopneas. The absolute changes in RR intervals relative to the beginning of the event during and after hypopneas are presented in subfigures (**c)** and (**d)**, respectively. In subfigures (**a)** and (**c)** the duration of every hypopnea event is normalized with its duration.

When comparing the within- and post-event RR intervals, the post-event RR intervals were shorter than within-event RR intervals regardless of the apnea or hypopnea duration (Tables 2 and 3). Moreover, the median difference between within- and post-event RR intervals increased with increasing respiratory event duration (Table 4, Figs. 1 and 2).

**Table 4:** The median differences between within-event (i.e. during a respiratory event) and post-event (i.e. within a 15-s segment following a respiratory event) RR intervals in different respiratory event duration groups.

	Apneas			Hypopneas		
	Men	Women	*p*-value	Men	Women	*p*-value
**10–20 s**						
*n* (%)	1699 (31.0)	953 (45.5)	** < 0.001**	6240 (35.9)	5966 (45.0)	** < 0.001**
ΔRR interval [ms]	48	30	** < 0.001**	32	23	** < 0.001**
**20–30 s**						
*n* (%)	2029 (37.0)	658 (31.4)	** < 0.001**	5816 (33.4)	4060 (30.6)	** < 0.001**
ΔRR interval [ms]	67	70	0.376	46	31	** < 0.001**
**Over 30 s**						
*n* (%)	1761 (32.1)	485 (23.1)	** < 0.001**	5348 (30.7)	3232 (24.4)	** < 0.001**
ΔRR interval [ms]	101	87	** < 0.001**	61	42	** < 0.001**

*p*-values presented in the table denote the statistical significance of difference in the median RR interval between men and women (the Mann–Whitney *U* test for continuous and the χ^2^-test for discrete variables). *p*-values denoting statistically significant differences (*p* < 0.01) are bolded. All presented median differences between within- and post-event RR intervals were significant (*p* < 0.001) according to Wilcoxon signed-rank test and all the differences between event duration derived groups were significant (*p* < 0.001) according to the Mann–Whitney *U* test. *ΔRR interval* the median differences between within- and post-event RR intervals, *n* the number of events used to determine the difference.

Effect sizes (Cohen’s *d*) for differences in HRV parameters between subgroups during and after the event are presented in Supplementary Tables S1–S6.

### HRV in women

The values of within-event RR interval SD, RMSSD, and pRR50 were higher in 20–30 s and > 30 s respiratory events compared to 10–20 s events (Tables 2 and 3). In apneas, the post-event average RR intervals, RMSSD, and pRR50 were greater in 20–30 s apneas than in 10–20 s and > 30 s apneas (Table 2). In hypopneas, the values of post-event RMSSD and pRR50 were the greatest in 20–30 s hypopneas with a statistically significant difference compared to 10–20 s and > 30 s hypopneas (Table 3). Furthermore, in both apneas and hypopneas, the relative difference between within- and post-event HRV parameter values decreased with increasing respiratory event duration (Tables 2 and 3).

**Figure 3:**
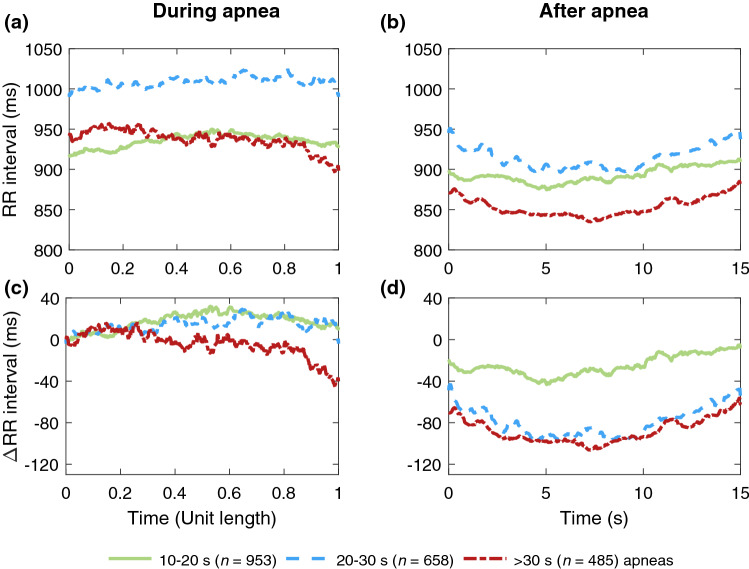
Median RR intervals of women (*n* = 232) (**a)** during apneas of different durations (*n* = 2 096) and (**b)** during a 15-s segment following apneas. The absolute change in RR intervals relative to the beginning of the event during and after apneas are presented in subfigures (**c)** and (**d)**, respectively. In subfigures (**a)** and (**c)** the duration of every apnea event is normalized with its duration.

**Figure 4:**
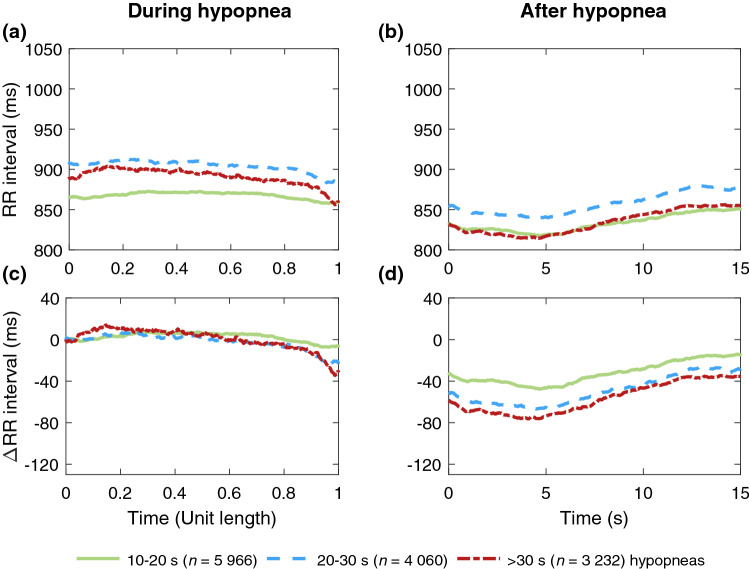
Median RR intervals of women (*n* = 359) (**a)** during hypopneas of different durations (*n* = 13 258) and (**b)** during a 15-s segment following hypopneas. The absolute change in RR intervals relative to the beginning of the event during and after hypopneas are presented in subfigures (**c)** and (**d)**, respectively. In subfigures (**a)** and (**c)** the duration of every hypopnea event is normalized with its duration.

The median within- and post-event RR intervals were the longest in 20–30 s respiratory events (Tables 2 and 3). Moreover, the post-event RR intervals were shorter than the within-event RR intervals (Tables 2 and 3) and the median difference between the within- and post-event RR intervals increased with increasing respiratory event duration (Table 4, Figs. 3 and 4).

Effect sizes (Cohen’s *d*) for differences in HRV parameters between subgroups during and after the event are presented in Supplementary Tables S1–S6.

## Discussion

This study investigated whether respiratory event-related changes in RR intervals and ultra-short-term HRV are modulated by the type and duration of the event and if these changes are sex-specific. We hypothesized that longer respiratory events cause greater ultra-short-term HRV than shorter events and decrease in RR interval after the events. We also hypothesized that these changes are greater in apneas and are emphasized in men. Consistent with our hypotheses, we found that longer respiratory events cause a larger decrease in RR interval values after the events and they generally cause greater ultra-short-term HRV than shorter events.

As hypothesized, longer respiratory events were associated with a greater median difference between within- and post-event RR intervals regardless of sex or event type (Table 4). These greater differences seemed to be caused by greater changes in post-event RR intervals rather than in within-event RR intervals, especially in apneas (Figs. 1 and 3), and this phenomenon was more emphasized in men (Figs. 1 and 2). Guilleminault et al.^23^ have shown similar cyclical heart rate variation, i.e. bradycardia during and tachycardia after the respiratory event. Longer apneas have been related to more severe bradycardia^24^ and a greater difference between within- and post-event RR intervals than shorter apneas^18^. Moreover, Chouchou et al*.*^25^ observed, consistently with our study, that the post-event RR interval shortening is greater with longer respiratory events. No oxygen desaturation is required to occur to score an apnea, and hypopnea can be scored with either ≥ 3% desaturation or arousal related to the airflow reduction^2^. Therefore, some of the analyzed respiratory events are not associated with desaturation. However, as 78% of apneas and 54% of hypopneas cause desaturation^26^, the degree of desaturation seems to be the most obvious explanation for the differences in RR intervals. This is in line with Kulkas et al.^19^ reporting that longer respiratory events are related to deeper desaturations that lead to greater SNS activation^11^. While some studies^18,23,24^ did not consider apneas and hypopneas separately, Chouchou et al*.*^25^ have shown that the type and duration of respiratory events do not significantly affect the decrease in RR intervals after the respiratory events. This finding seems counterintuitive to data supporting differences between apneas and hypopneas and may be a consequence of the small sample size (*n* = 16). It is clear, that the connection between HRV and desaturations warrants further research.

The ultra-short-term HRV was generally higher with longer respiratory events regardless of the respiratory event type, supporting our hypotheses. However, the relative difference between within- and post-event HRV parameter values decreased with increasing respiratory event duration. The long-term intermittent hypoxemia increases the chemosensitivity of the carotid body leading to increased sympathetic activity and heart rate^27^, and thus, reduced long-term HRV. Furthermore, short respiratory events reflect higher arousability causing sleep fragmentation linked to elevated mortality in both sexes^28,29^. Moreover, the risk of arrhythmias is markedly increased in OSA^12^, especially immediately after respiratory events^30^. These findings^12,15,27–30^ imply that considering HRV together with the respiratory event characteristics in the diagnosis of OSA could be useful when assessing the cardiac consequences of OSA in a more detailed manner. However, our results are based on respiratory events pooled only by their duration and have not considered the AHI or other OSA severity markers in patients. Since the regulation of SNS and PNS activity is individual, the combined effect of respiratory event duration and OSA severity on HRV needs further investigation.

As hypothesized, apneas caused greater differences between within- and post-event median RR intervals than hypopneas in both men and women (Table 4). Similarly, the ultra-short-term HRV was also greater in apneas compared to hypopneas (Tables 2 and 3). The RR interval reached its minimum after the respiratory event later in apneas (~ 7 s, Figs. 1 and 3) than in hypopneas (~ 4 s, Figs. 2 and 4). Apneas cause deeper oxygen desaturations than hypopneas^19^ and severe desaturations are a significant risk factor for cardiovascular diseases^20,21^. In OSA, the frequency and severity of the respiratory events, especially apneas, increase towards morning^31^, and the probability of cardiogenic sudden deaths is elevated between midnight and 6 a.m.^32^. In addition, it has been shown that the risk of arrhythmia is markedly increased shortly after a respiratory event^30^. Negative intrathoracic pressure, hypoxemia-induced pulmonary hypertension, and increased sympathetic tone have been proposed to explain the increased propensity for arrhythmias in patients with OSA^33^. In this study, HRV was measured from a relatively low number of beats: respiratory event induced arrhythmias may also manifest as increased ultra-short-term HRV parameter values. Higher ultra-short-term HRV could be more harmful due to increased beat-to-beat variation within a very short time, although low long-term HRV is more commonly associated with poor health^7,14^. Together with other findings^19–21,30–33^, our results indicate that apneas and hypopneas affect cardiac regulation differently. Detailed ECG and HRV analysis could be a useful tool in addition to AHI in OSA severity assessment, especially for patients with cardiovascular diseases, to help assess the risk of cardiovascular consequences.

It has been shown that long-term HRV is significantly lower in women compared to men and it is characterized by a relative dominance of PNS activity despite shorter RR intervals^22^. In hypopneas, values of all ultra-short-term HRV parameters were significantly higher in men compared to women (Table 3). However, there were inconsistencies in the values of HRV parameters related to apneas (Table 2). In apneas, the within- and post-event SD values were greater in men. The RMSSD and pRR50 values of women were generally equal to or greater than those of men but these differences were not statistically significant. In addition, the differences between within- and post-event RR intervals were generally greater in men than in women (Table 4). Longer respiratory events cause deeper desaturations, and apneas lead to deeper desaturations than hypopneas^19^. It remains unclear whether the ultra-short-term HRV was affected more by the respiratory event severity or the sex since in apneas, the differences between sexes were not consistent. However, men have been reported to have more and longer respiratory events with deeper oxygen desaturations than women^34,35^. Men could be exposed to more severe cyclical heart rate variation due to intermittent desaturations causing greater differences in average RR interval compared to women, which could partially explain our results. Based on previous literature and this study, it is evident that men and women should be studied separately when analyzing HRV of OSA patients.

The main limitation of this study is the use of only time-domain HRV parameters as this prohibits assessing the vagal and sympathetic tones of ANS or the spectral variations of HRV precisely. However, frequency-domain HRV parameters require a longer RR interval segment for analysis^3^ and are thus incompatible with ultra-short-term HRV. The use of ultra-short-term HRV measurements and RR interval segments instead of short-term measurements and segments is also a limitation of the present study. Currently, there are no normative results for ultra-short-term HRV because the short- and long-term measurements are more often used^3,4^, but the use of ultra-short-term HRV analysis has shown promise^3,5^. In addition, minimum ultra-short-term periods of 30 s and 60 s for RMSSD and pRR50, respectively, have been suggested for healthy subjects^5^. In our analyses comprising OSA patients, even shorter RR interval segments were used as the minimum respiratory event duration used for within-event segments was 10 s. Moreover, the post-event segment duration of 15 s was chosen to reliably present temporal changes in RR intervals and not to excessively exclude respiratory events. Importantly, ultra-short-term HRV enables the assessment of HRV during and after individual respiratory events. This would not be possible with longer short-term periods.

Although the automated R peak detection does not recognize irregular heart rhythms^36^, which can be considered as a limitation, it enables effective and reproducible analysis. Another limitation of our study is excluding a substantial number of respiratory events (*n* = 61,638, 62% of all events), whose effects we were not able to study. All successive respiratory events having less than 15 s between them were excluded. This exclusion, however, enabled the evaluation of the post-event RR interval recovery to its pre-event level without the next event interfering with the recovery. Moreover, we included all three types of respiratory events (obstructive, central, and mixed) and did not differentiate between them. This can be considered as a limitation due to their potentially different effects on SNS and PNS activities.

In addition, we acknowledge including the patients with a history of multimorbidity, such as hypertension or diabetes, as a limitation, since several comorbidities may affect the ultra-short-term HRV in addition to OSA. Not considering the effects of sleep stages or respiratory event-related arousals is also a limitation. It has been reported that RR intervals shorten in the proximity of arousal in both healthy subjects^37^ and OSA patients^38^, and our results show a similar pattern (Figs. 1, 2, 3, 4). Furthermore, several studies have shown increased PNS activity and higher short-term HRV during non-rapid eye movement sleep and lower HRV with increased SNS activity in rapid eye movement sleep^37,39^, with sleep stages modulating HRV more strongly than OSA^40^. Thus, further studies simultaneously investigating the effect of arousals, sleep stages, desaturations, HRV, and respiratory events are warranted.

To conclude, as the changes in RR interval and ultra-short-term HRV reflect immediate physiological consequences, this study provides valuable insight into cardiovascular stress associated with apneas and hypopneas. Our results show that higher ultra-short-term HRV and greater variation between within- and post-event RR intervals are more strongly related to longer respiratory event duration, apneas, and male sex. The type and duration of a respiratory event affect the heart rate and HRV. This study demonstrates that cardiac and respiratory event characteristics may provide valuable information in addition to the AHI when diagnosing sleep apnea. These results and limitations also highlight the need for further prospective studies considering the connection between respiratory event severity, desaturations, and HRV.

## Methods

### PSG data

The dataset used in this study comprised 892 full diagnostic PSGs of patients with suspected OSA. PSGs were conducted at the Princess Alexandra Hospital (Brisbane, Australia) during 2015–2017 using the Compumedics Grael acquisition system (Compumedics, Abbotsford, Australia) and they were retrospectively analysed. Patients without any respiratory events (apneas or hypopneas, *n* = 24) or with incomplete demographic data (*n* =  6) were excluded. Demographic data was considered incomplete if the medical records had no details of the existence of comorbidities listed in Table 1 or the patient’s smoking habits. Since previous cardiovascular diseases may affect the normal functioning of the heart, we excluded patients with a history of cardiac arrhythmias and/or known heart failure (*n* = 104). Therefore, the final dataset comprised PSGs of 758 patients (Table 1). Every recording was scored manually by experienced scorers in compliance with the prevalent AASM 2012 guidelines^2^. Approval for retrospective data collection was obtained from the Institutional Human Research Ethics Committee of the Princess Alexandra Hospital (HREC/16/QPAH/021 and LNR/2019/QMS/54313). All procedures performed in studies involving human participants were in accordance with the ethical standards of the institutional and/or national research committee and with the 1964 Helsinki declaration and its later amendments or comparable ethical standards. The need for informed consent was waived by the Metro South Human Research Ethics Committee due to retrospective nature of the study.

### HRV analysis

The nocturnal ECGs were recorded using the modified lead II^2^ with a sampling frequency of 256 Hz. Smoothness priors method^41^ was used to detrend the ECGs using a smoothing parameter λ value of 500. The Pan-Tompkins method^36^ was used to detect the R-peaks from the ECGs. The data analysis was performed with MATLAB R2018b (MathWorks Inc, MA, USA).

Within- and post-event segments were separated from the ECG signal for each scored respiratory event. The within-event segment was defined from the start to the end of the respiratory event. The post-event segment was the period of 15 s immediately after the respiratory event. A post-event duration of 15 s was chosen to reliably present temporal changes in RR intervals without excessively limiting the number of events. No overlapping post- and within-event segments of consecutive respiratory events were allowed. We excluded both consecutive respiratory events having less than 15 s between them, i.e. with overlapping post- and within-segments (*n* = 61,638). Finally, the remaining 38,247 respiratory events (7585 apneas and 30,662 hypopneas) were included for HRV analysis. Apneas and hypopneas were divided into three subgroups based on their duration separately for men and women: 10–20 s events, 20–30 s events, and > 30 s events (Table 1). All event types (obstructive, mixed, and central) were included in the analysis.

For each ECG segment, the average RR interval and HRV parameters consisting of the standard deviation (SD) of RR intervals, the root mean square of successive differences (RMSSD), and the proportion of adjacent RR intervals differing more than 50 ms (pRR50) were determined^3^. The time-domain parameters were chosen instead of the frequency-domain parameters due to their better suitability for ultra-short-term HRV analysis^3,5^. The median difference in RR intervals between within- and post-event segments was calculated from the average RR intervals by subtracting the post-event RR interval from the within-event RR interval. The statistical significance was evaluated with the Wilcoxon signed-rank test when comparing within- and post-event HRV parameters and with the Mann–Whitney *U* test when comparing the HRV parameters between the event duration-based subgroups. Due to large sample sizes and multiple testing, we have used a significance level of *p* < 0.001 for demographic differences (Table 1) and *p* < 0.01 for HRV analyses (Tables 2, 3, and 4). Cohen’s *d* was used to evaluate the effect sizes for differences in HRV parameters between subgroups during and after the respiratory events.

The change in RR intervals was illustrated as a function of time. For illustrative reasons, the RR interval sequences were detrended with smoothness priors method and spline interpolated during and after the respiratory events. However, the interpolated RR interval values under 200 ms were excluded due to the 200 ms refractory period between two successive QRS complexes^36^.

## Supplementary information

Supplementary Information.

## Data Availability

The data includes medical records and personal information and therefore, the data can only be shared within the confinements of the Australian legislation and ethical conventions. Reasonable requests considering data sharing will be individually assessed.
