# Supplementary material for: Longer apneas and hypopneas are associated with greater ultra-short-term HRV in obstructive sleep apnea
Source: Sci Rep. 2020 Dec 9;10:21556. doi: 10.1038/s41598-020-77780-x (PMC7726571; doi:10.1038/s41598-020-77780-x)
Supplement: Supplementary file 1 — Supplementary Information. [file 41598_2020_77780_MOESM1_ESM.pdf]

Salla Hietakoste, Henri Korkalainen, Samu Kainulainen, Saara Sillanmäki, Sami Nikkonen, Sami Myllymaa, Brett Duce, Juha Töyräs, and Timo Leppänen

Effect sizes evaluated by Cohen's *d* for differences between subgroups

Table S1. Within-event HRV parameter effect sizes (Cohen's *d*) for differences between apnea duration (s) subgroups.

	**Men**			**Women**			**Men vs. Women**		
	**10–20 s vs. 20–30 s**	**10–20 s vs. >30 s**	**20–30 s vs. >30 s**	**10–20 s vs. 20–30 s**	**10–20 s vs. >30 s**	**20–30 s vs. >30 s**	**10–20 s**	**20–30 s**	**>30 s**
**RR**	0.04	0.13	0.09	0.43	0.27	0.11	0.02	0.37	0.12
**SD**	0.24	0.32	0.17	0.29	0.43	0.12	0.11	0.12	0.19
**RMSSD**	0.21	0.25	0.10	0.29	0.38	0.07	0.06	0.05	0.11
**pRR50**	0.27	0.35	0.08	0.36	0.52	0.19	0.01	0.04	0.13

HRV = heart rate variability, RR = RR interval, SD = standard deviation of RR intervals, RMSSD = root mean square of successive differences, and pRR50 = the number of adjacent RR intervals differing more than 50 ms divided by the total number of RR intervals.

Table S2. Within-event HRV parameter effect sizes (Cohen's *d*) for differences between hypopnea duration (s) subgroups.

	**Men**			**Women**			**Men vs. Women**		
	**10–20 s vs. 20–30 s**	**10–20 s vs. >30 s**	**20–30 s vs. >30 s**	**10–20 s vs. 20–30 s**	**10–20 s vs. >30 s**	**20–30 s vs. >30 s**	**10–20 s**	**20–30 s**	**>30 s**
**RR**	0.27	0.35	0.07	0.22	0.15	0.08	0.24	0.30	0.46
**SD**	0.15	0.26	0.10	0.08	0.13	0.02	0.08	0.09	0.19
**RMSSD**	0.08	0.15	0.06	0.06	0.07	0.00	0.06	0.06	0.13
**pRR50**	0.08	0.15	0.07	0.09	0.06	0.03	0.10	0.09	0.20

HRV = heart rate variability, RR = RR interval, SD = standard deviation of RR intervals, RMSSD = root mean square of successive differences, and pRR50 = the number of adjacent RR intervals differing more than 50 ms divided by the total number of RR intervals.

Table S3. Within-event HRV parameter effect sizes (Cohen's *d*) for differences between apnea and hypopnea duration (s) subgroups.

	**Men**			**Women**		
	**10–20 s**	**20–30 s**	**>30 s**	**10–20 s**	**20–30 s**	**>30 s**
**RR**	0.19	0.04	0.01	0.42	0.60	0.50
**SD**	0.11	0.24	0.27	0.09	0.20	0.36
**RMSSD**	0.00	0.16	0.19	0.01	0.17	0.28
**pRR50**	0.13	0.07	0.09	0.04	0.21	0.41

HRV = heart rate variability, RR = RR interval, SD = standard deviation of RR intervals, RMSSD = root mean square of successive differences, and pRR50 = the number of adjacent RR intervals differing more than 50 ms divided by the total number of RR intervals.

Table S4. Post-event HRV parameter effect sizes (Cohen's *d*) for differences between apnea duration (s) subgroups.

	**Men**			**Women**			**Men vs. Women**		
	**10–20 s vs. 20–30 s**	**10–20 s vs. >30 s**	**20–30 s vs. >30 s**	**10–20 s vs. 20–30 s**	**10–20 s vs. >30 s**	**20–30 s vs. >30 s**	**10–20 s**	**20–30 s**	**>30 s**
**RR**	0.04	0.26	0.19	0.21	0.04	0.21	0.12	0.32	0.28
**SD**	0.16	0.20	0.00	0.26	0.31	0.09	0.09	0.06	0.00
**RMSSD**	0.15	0.18	0.02	0.28	0.33	0.08	0.02	0.01	0.07
**pRR50**	0.16	0.14	0.02	0.24	0.13	0.10	0.01	0.07	0.00

HRV = heart rate variability, RR = RR interval, SD = standard deviation of RR intervals, RMSSD = root mean square of successive differences, and pRR50 = the number of adjacent RR intervals differing more than 50 ms divided by the total number of RR intervals.

Table S5. Post-event HRV parameter effect sizes (Cohen's *d*) for differences between hypopnea duration (s) subgroups.

	Men			Women			Men vs. Women		
	**10–20 s vs. 20–30 s**	**10–20 s vs. >30 s**	**20–30 s vs. >30 s**	**10–20 s vs. 20–30 s**	**10–20 s vs. >30 s**	**20–30 s vs. >30 s**	**10–20 s**	**20–30 s**	**>30 s**
**RR**	0.18	0.13	0.05	0.15	0.00	0.16	0.15	0.20	0.30
**SD**	0.06	0.10	0.04	0.05	0.05	0.00	0.07	0.09	0.13
**RMSSD**	0.04	0.05	0.01	0.03	0.02	0.02	0.05	0.06	0.09
**pRR50**	0.07	0.10	0.03	0.11	0.00	0.11	0.12	0.08	0.21

HRV = heart rate variability, RR = RR interval, SD = standard deviation of RR intervals, RMSSD = root mean square of successive differences, and pRR50 = the number of adjacent RR intervals differing more than 50 ms divided by the total number of RR intervals.

Table S6. Post-event HRV parameter effect sizes (Cohen's *d*) for differences between apnea and hypopnea duration (s) subgroups.

	Men			Women		
	**10–20 s**	**20–30 s**	**>30 s**	**10–20 s**	**20–30 s**	**>30 s**
**RR**	0.11	0.11	0.29	0.38	0.43	0.29
**SD**	0.09	0.17	0.18	0.08	0.27	0.33
**RMSSD**	0.04	0.15	0.16	0.06	0.29	0.36
**pRR50**	0.08	0.17	0.12	0.23	0.33	0.33

HRV = heart rate variability, RR = RR interval, SD = standard deviation of RR intervals, RMSSD = root mean square of successive differences, and pRR50 = the number of adjacent RR intervals differing more than 50 ms divided by the total number of RR intervals.
